# Understanding AP2/ERF Transcription Factor Responses and Tolerance to Various Abiotic Stresses in Plants: A Comprehensive Review

**DOI:** 10.3390/ijms25020893

**Published:** 2024-01-11

**Authors:** Ziming Ma, Lanjuan Hu, Wenzhu Jiang

**Affiliations:** 1 Jilin Provincial Engineering Laboratory of Plant Genetic Improvement, College of Plant Science, Jilin University, Changchun 130062, China; hulj@jlu.edu.cn; 2 Max-Planck-Institute of Molecular Plant Physiology, Am Muehlenberg 1, 14476 Potsdam-Golm, Germany; 3 Plant Genetics, TUM School of Life Sciences, Technical University of Munich (TUM), Emil Ramann Str. 4, 85354 Freising, Germany

**Keywords:** AP2/ERF transcription factor, abiotic stress, target gene, hormone signalling

## Abstract

Abiotic stress is an adverse environmental factor that severely affects plant growth and development, and plants have developed complex regulatory mechanisms to adapt to these unfavourable conditions through long-term evolution. In recent years, many transcription factor families of genes have been identified to regulate the ability of plants to respond to abiotic stresses. Among them, the AP2/ERF (APETALA2/ethylene responsive factor) family is a large class of plant-specific proteins that regulate plant response to abiotic stresses and can also play a role in regulating plant growth and development. This paper reviews the structural features and classification of AP2/ERF transcription factors that are involved in transcriptional regulation, reciprocal proteins, downstream genes, and hormone-dependent signalling and hormone-independent signalling pathways in response to abiotic stress. The AP2/ERF transcription factors can synergise with hormone signalling to form cross-regulatory networks in response to and tolerance of abiotic stresses. Many of the AP2/ERF transcription factors activate the expression of abiotic stress-responsive genes that are dependent or independent of abscisic acid and ethylene in response to abscisic acid and ethylene. In addition, the AP2/ERF transcription factors are involved in gibberellin, auxin, brassinosteroid, and cytokinin-mediated abiotic stress responses. The study of AP2/ERF transcription factors and interacting proteins, as well as the identification of their downstream target genes, can provide us with a more comprehensive understanding of the mechanism of plant action in response to abiotic stress, which can improve plants’ ability to tolerate abiotic stress and provide a more theoretical basis for increasing plant yield under abiotic stress.

## 1. Introduction

Plants are often affected by biotic or abiotic stresses during growth and development. Biotic stress is the stress of various biological factors unfavourable to the survival and development of plants, mainly due to infection and competition, such as diseases, insect pests, and weed damage. Abiotic stresses are environmental conditions unfavourable to plant survival and development and even lead to injury, damage, and death. Abiotic stresses include drought, high salt, low temperature, high temperature, nutrient stress, and heavy metals [1,2,3]. With global climate change, the impact of these stresses on plants is increasing. It has caused the reduction of global production of major crops and ultimately has a serious impact on global food security [4,5]. This paper reviews the structural features and classification of AP2/ERF transcription factors that are involved in transcriptional regulation, reciprocal proteins, downstream genes, and hormone-dependent signalling and hormone-independent signalling pathways in response to abiotic stress. In the future, we can focus on studying AP2/ERF transcription factors as a signal regulatory gene network in response to abiotic stress to improve plant tolerance to abiotic stress from a signalling perspective and increase crop yields.

Abiotic stress negatively impacts plant growth and development, directly threatening plant survival and yield [6,7]. Plants have evolved a series of complex response mechanisms for normal growth in unfavourable environments, such as through the perception of abiotic stress signals, hormone-dependent signalling and hormone-independent signalling channel signal transduction, induction of abiotic stress gene expression, and further activation of physiological and metabolic responses [7,8,9]. The gene products of plants in response to abiotic stresses are divided into two categories: The first type is regulatory proteins, which consist of protein factors that regulate downstream signal transduction and the expression of abiotic stress-responsive genes. The second type is functional proteins, which directly affect plant adaptation to abiotic stresses [10]. Regulatory proteins include protein kinases, phosphokinases, and transcription factors (AP2, NAC, MYB, WRKY, and bZIP transcription factors) [11,12,13]. Functional proteins include proline, soluble sugars, late embryogenesis abundant protein (LEA), dehydrins, superoxide dismutase (SOD), peroxidase (POD) and catalase (CAT), water channel proteins, etc. These proteins can mitigate damage to plant cells from various abiotic stresses by maintaining plant cell expansion pressure, scavenging reactive oxygen species, and protecting the structure of intracellular biomolecules [14,15].

Transcription factors are proteins that regulate downstream gene expression by binding to specific sequences in DNA or other protein complexes. Transcriptional regulation refers to the combination of transcription factors with cis-acting elements upstream of stress-responsive genes to activate or repress gene expression during the process of gene expression [16,17]. Abiotic stresses such as drought, low temperature, and high salt lead to an increase in abscisic acid (ABA) biosynthesis, which activates the binding of transcription factors to cis-acting elements in specific sequences in the promoter regions of the corresponding downstream stress-responsive genes, regulating gene expression and, thus, modulating plant tolerance in response to abiotic stresses [18,19]. As one of the plant-specific transcription factor families, the AP2/ERF family of transcription factors is of great significance for plant survival and development. Nowadays, with the release of the whole genome sequences of many plants, their functional regulatory networks in crops such as rice (*Oryza sativa* L.), maise (*Zea mays* L.), and soybean (*Glycine max* L.) will be gradually revealed. This article discusses the structural features of AP2/ERF transcription factors, including their binding elements, regulation of transcription, and interaction with other proteins. It also discusses the progress of research on the role of AP2/ERF in regulating the response to abiotic stresses and provides references for future research on AP2/ERF transcription factors to improve plants’ ability to cope with abiotic stresses [20,21,22].

### 1.1. Classification and Structural Classification of AP2/ERF Transcription Factors

**Figure 1:**
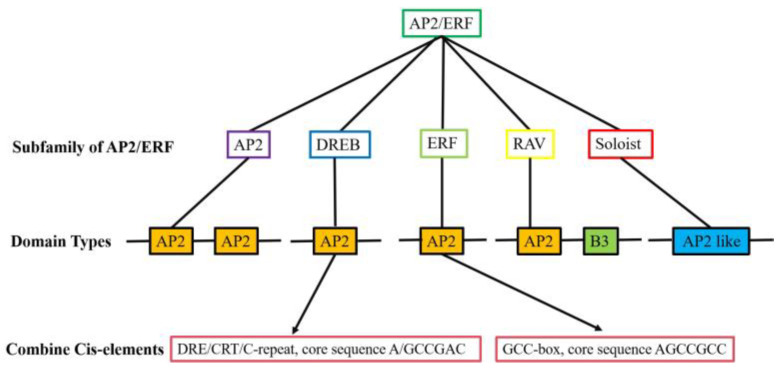
AP2/ERF transcription factor family classification and structural features. They can be divided into five main groups based on the number of AP2/ERF structural domains, namely AP2 (APETALA2), DREB (dehydration-responsive element binding protein), ERF (ethylene-responsive factor), RAV (related to ABI3/VP1), and Soloist. The AP2 domain contains two AP2 structural domains; the DREB and ERF domains contain an AP2 structural domain; the RAV domain contains an AP2 structural domain and a B3 structural domain; and the Soloist domain contains an AP2-like structural domain. The ERF subfamily of transcription factor members can participate in the regulation of abiotic stress by binding to the ethylene response element (ERE) (GCC-box, core sequence AGCCGCC). Members of the DREB subfamily of transcription factors specifically recognise and bind to a DRE/CRT element (dehydration responsive element/C-repeat, core sequence A/GCCGAC) in the promoter region of another gene. The yellow background represents the AP2 domain, the green background represents the B3 domain, and the blue background represents the AP2 like domain.

The AP2/ERF transcription factor family is a large group of plant-specific transcription factors that regulate plant growth, development, and abiotic stress response. Many AP2/ERF family genes were identified in the plant genome. They can be divided into five main groups based on the number of AP2/ERF structural domains, namely AP2 (APETALA2), ERF (ethylene-responsive factor), DREB (dehydration-responsive element binding protein), Soloist, and RAV (related to ABI3/VP1) (Supplementary Table S1). For example, AP2/ERF transcription factor genes, namely *Dehydration responsive factor 2B* (*OsDREB2B*), *Reduced plant height 1* (*OsRPH1*), *Ethylene response AP2/ERF factor* (*OsEATB*), and *APETALA-2-Like transcription factor gene 39* (*OsAP2-39*), are able to affect rice growth and development, and consequently plant height, by regulating the expression of Gibberellin (GA) metabolic genes [23,24,25,26]. Present-day studies are more extensive and in-depth for the AP2, DREB, and ERF subfamilies, whereas very few studies have been reported on the Soloist subfamily, whose nucleotide sequences are known to be highly conserved in most plants [27] (Figure 1). The structure of AP2/ERF transcription factors is characterised by four major functional regions, namely the DNA-binding domain, the transcription regulation domain, the oligomerisation site, and the nuclear localisation signal (NLS), and the AP2/ERF binding domain is highly conserved [28]. The AP2/ERF transcription factor structural domain contains 60–70 amino acid residues, forming a typical three-dimensional structure according to three β-folds and one α-helix. The YRG and RAYD elements in the AP2 domain play an important role in DNA binding activity. The YRG element at the N-terminal end of the domain consists of approximately 19–22 hydrophilic amino acid residues and promotes DNA binding through bases and hydrophilic groups. The RAYD element at the C-terminal end of the domain consists of about 42–43 residues and mediates protein–protein interactions through the α-helices or interacts with DNA through the hydrophobic surface of the α-helices [29]. Many studies have shown that AP2/ERF transcription factors are highly structurally similar, which may lead to a large amount of gene redundancy in the AP2/ERF family of transcription factors [23,24].

### 1.2. Cis-Acting Elements Recognized by AP2/ERF Transcription Factors

In previous promoter region analyses of AP2/ERF transcription factor-regulated genes, scientists have identified many AP2/ERF transcription factors capable of specifically binding to stress-responsive gene initiator cis-elements in the promoter region [30,31,32]. The ERF subfamily of transcription factor members can participate in the regulation of abiotic stress by binding to the ethylene-response element (ERE) (GCC-box, core sequence AGCCGCC). Members of the DREB subfamily of transcription factors specifically recognise and bind to a DRE/CRT element (dehydration-responsive element/C-repeat, core sequence A/GCCGAC) in the promoter region of another gene, regulating the expression of response genes related to drought, low temperature, and salt stress [33,34,35,36]. The AP2/ERF transcription factors bind not only to both DRE/CRT and ERE elements but also to other cis-elements, such as coupling element 1 (CE1, TGCCACCG), coupling element 3 (CE3, CGCG) and hypoxia-responsive promoter element (HRPE). The AP2/ERF transcription factors also bind to these cis-elements, for example, CAACA, ATCTA, CATGCA, CGNCCA, and ATCGAG [37,38,39,40].

## 2. AP2/ERF Transcriptional Regulation and Interacting Protein under Abiotic Stresses

### 2.1. AP2/ERF Transcriptional Regulation under Abiotic Stresses

Under abiotic stresses, some genes related to adversity in plants can bind to cis-acting elements conserved in AP2/ERF transcription factors to regulate the expression of their transcription factor gene, which include low-temperature responsive elements, heat shock-responsive elements, and ABA-responsive elements. It has been shown that heat shock factor 1 (HSF1) and ABA-responsive element binding protein (ABRE) can bind to the heat shock response element in the *dehydration responsive factor 2A* (*DREB2A*) promoter and regulate the expression of their respective transcription factor gene, thereby altering the plant’s ability to tolerate abiotic stresses [41,42,43,44]. Song et al. [45] showed that the interaction of the APETALA2/EREBP-type transcription factor 7 (AtERF7) with the protein kinase S3 (PKS3) is involved in the regulation of the plant ABA response. AtERF7 binds to the GCC box and is able to inhibit gene transcription. AtERF7 interacts with the transcriptional corepressor AtSin3, which in turn may interact with the histone deacetylase 19 (HDA19). HDA19 and SIN3-like 3 (AtSin3) enhance the transcriptional repression activity of AtERF7. Thus, AP2/ERF transcription can be regulated by histone modifications such as phosphorylation, ubiquitination, methylation, and acetylation by modulating the spatial state of chromatin [46,47,48,49]. Kavas et al. [50] identified 180 AP2/ERF superfamily genes in *Phaseolus vulgaris*. MicroRNA target transcript analyses identified in computer simulations identified almost all PvAP2-ERF genes as MicroRNA targets in 44 different plant species; the most abundant target gene was the *AP2-ERF transcription factor gene* (*PvERF20*, *PvERF25*, *PvERF62*, *PvERF78*, *PvERF113*, and *PvERF173*).

### 2.2. AP2/ERF Transcription Factors Interacting Protein under Abiotic Stresses

Transcription factor (TF), also known as trans-acting factor, is a DNA-binding protein that specifically interacts with cis-acting elements of eukaryotic genes and activates or inhibits gene transcription. Many studies have shown that AP2/ERF transcription factors interact with other transcription factors to form protein complexes or can directly bind to the promoters of their downstream target genes to repress or promote gene expression [51,52]. AP2/ERF family transcription factor OsDREB2B was able to regulate the expression of *OsAP2-39* by binding to its promoter, and OsDREB2B interacted with OsWRKY21 to regulate the expression of GA metabolism genes and inhibit GA synthesis, leading to a decrease in GA content and, thus, exerting a negative effect on rice growth and development [23]. The AP2/ERF transcription factor *OsRPH1* overexpression resulted in reduced plant height, and OsRPH1 interacted with the blue light receptor Cryptochrome 1 (OsCRY1b) [24]. Tiwari et al. [53] identified a short “EDLL” motif that is present in ethylene response factor 98 (AtERF98)/TDR1 and other branch members from the same AP2 subfamily. This motif has a unique arrangement of acidic amino acids and hydrophobic leucines and functions as a strong activation domain that partially overcomes the repression conferred by the homeobox protein 2 (AtHB2) transcription factor, which contains an ERF-associated amphiphilic repressor (EAR) motif. Overexpression of *ethylene response factor 7* (*AtERF7*) plants has reduced the sensitivity of defence cells to ABA and increased water loss. Ethylene response factor 3 (ERF3) interacts with the subunit of the histone deacetylase complex SAP18 (SIN3-associated polypeptide P18) and co-recruits histone deacetylase 19 (HDA19) to form a complex which inhibits the expression of the associated genes [54]. Franco-Zorrilla et al. [55] analysed co-regulated gene and transcriptome data from transcription factor mutants. The results indicate that at least one target sequence for each transcription factor is functionally important and that the function of a transcription factor as an activator or a repressor can be predicted from specific DNA sequences. Therefore, genes co-regulated by AP2/ERF transcription factors are also enriched for AP2/ERF target genes, and future analyses of homologous sequences by scientists will help to identify putative targets of transcription factors and predict their biological functions.

## 3. AP2/ERF by Participating in the Regulation of Hormone-Mediated Abiotic Stresses

Phytohormones, also known as plant natural hormones or plant endogenous hormones, refer to a number of organic compounds produced by the plant body in minute quantities that can regulate (promote, inhibit) their own physiological processes. The main role of phytohormones is to regulate the ability of plants to resist the adverse external environment and plant growth and development. Studies have shown that AP2/ERF transcription factors can synergy with hormone signalling to form a cross-regulatory network, e.g., by participating in growth and development and abiotic stress responses mediated by the plant hormones abscisic acid (ABA), gibberellin (GA), auxin (IAA), ethylene (ET), brassinosteroid (BR) and cytokinin (CTK) [56,57,58,59,60].

### 3.1. AP2/ERF Transcription Factors Involved in ABA-Mediated Stress Response

The main role of ABA is to promote the shedding of organs such as plant leaves, fruits, and calyxes and to regulate plant growth and development. ABA is one of the key hormones in response to abiotic stresses such as drought, salt, cold, and heat. ABA promotes the synthesis of osmotic substances and protects against abiotic stresses by regulating stomatal opening, closure, and root architecture [61,62]. Overexpression of *ethylene response factor 71* (*OsERF71*) plants reduced water loss, resulting in increased tolerance to drought stress. OsERF71 was able to regulate the expression of ABA-responsive and proline biosynthesis genes under drought stress, resulting in increased sensitivity to exogenous ABA treatment and proline accumulation [63]. Cheng et al. [64] identified 229 AP2/ERF genes in the latest maise reference genome, of which 32 ZmAP2/ERFs regulate biotic stress and 24 ZmAP2/ERFs are involved in abiotic stress response. *Dehydration responsive factor* (*ZmDREB39*) and *ZmDREB89* were up-regulated in response to ABA. Xiong et al. [65] identified a total of 135 TkERF genes from *Trichosanthes kirilowii*. A co-expression network constructed using transcriptome data from different flowering stages showed that 67 of the AP2/ERF genes were associated with abscisic acid signalling pathway members. Seventeen genes were found to be up-regulated when tissue culture seedlings were treated with ABA, suggesting that some members of the TkERF gene family may be involved in phytohormone signalling pathways. Twenty genes were up-regulated under PEG treatment, suggesting that these selected genes may be involved in plant drought stress. AP2 transcription factor 10 (TaAP2-10) is able to be induced by abiotic stress and ABA hormone treatment [66]. The CBF (C-repeat binding factor) subfamily of transcription factors belongs to the AP2/ERF (Apetala 2/ethylene response factor) family of transcription factors. Research shows cis-acting elements in the promoter region of BpCBFs that are associated with environmental stress and hormones. Most of these transcription factors were found to be responsive to ABA or salt stress in different plant tissues after treatment using ABA or salt [67].

### 3.2. AP2/ERF Transcription Factors Involved in GA-Mediated Stress Response

The main physiological role of GA is to promote the elongation of plant cells, increase plant height, promote growth and development of lateral buds, promote flower bud differentiation and flowering, and inhibit ageing and shedding of leaves. Li et al. [68] identified a total of 218 AP2/ERF genes in the sugarcane genome, and the presence of multiple cis-regulatory elements (CREs) in the SsAP2/ERF promoter was associated with abiotic stress, suggesting that SsAP2/ERF activity may contribute to the adaptation of sugarcane to environmental changes. *Soloist subfamily transcription factor 4* (*SsSoloist4*) was most significantly up-regulated in response to treatment with the exogenous hormone GA, suggesting that this gene may play a role in GA-associated response. OsDREB2B is an AP2/ERF family transcription factor; overexpression of *OsDREB2B* in rice can significantly improve rice drought tolerance [69]. Ma et al. [23] found that *OsDREB2B* overexpression resulted in shorter plant height, and the length of the second leaf sheath in overexpressed plants was restored to that of the wild type by exogenous GA_3_ application. Expression of GA biosynthesis genes was altered in overexpression *OsDREB2B* plants. Fu et al. [70] found that overexpression of *AP2/EREBP transcription factor 20* (*ZmEREB20*) in *Arabidopsis* enhanced ABA sensitivity and caused delayed seed germination under salt stress by regulating ABA and GA-related genes. Zhang et al. [71] found that approximately 49 genes containing complete AP2/ERF structural domains were identified from the Taxus x media (Yew tree) transcriptome database. Nine of these TmERF genes could respond to low temperature and hormone treatments, and the expression of the *AP2/ERF transcription factor gene* (*TmERF5*, *TmERF14*, and *TmERF36*) was elevated under GA treatment. These findings indicate their potential involvement in crosstalk between abiotic stress response signalling pathways.

### 3.3. AP2/ERF Transcription Factors Involved in IAA-Mediated Stress Response

The main effects of IAA are to promote the division and elongation of plant cells, the growth of roots and stems, the development and ripening of fruits, and the regulation of plants’ form and growth direction. Cai et al. demonstrated a regulatory role for APETALA2/ethylene response factor (AP2/ERF) transcription factor 96 (OsERF096) in the cold stress response. Targeted metabolomics analyses revealed that OsERF096 can respond to cold stress by regulating IAA accumulation and signalling [72]. Huang et al. [73] found that *AP2/ERF transcription factor 12* (*ERF012*) overexpression showed resistance to temperature, drought, salt, and heavy metal stresses. Overexpression *ERF012* inhibited root growth and promoted root hair development and leaf senescence. The application of exogenous IAA effectively mitigated this effect. ERF012 may down-regulate its target genes *Cinnamate-4-hydroxylase* (*AtC4H*) and *4-coumarate CoA ligase 1* (*At4CL1*), resulting in reduced IAA accumulation leading to leaf senescence. Overexpression *AP2/ERF transcription factor B1* (*SlERF.B1*) showed significantly higher sensitivity to salt treatment at both phenotypic and physiological levels. Plants that overexpressed *SlERF.B1* showed low tolerance to mannitol and drought stresses. *SlERF.B1* expression was induced by salt, mannitol, cold, heat, and ACC treatments but was inhibited by ABA, IAA, and 1-MCP treatments [74].

### 3.4. AP2/ERF Transcription Factors Involved in ET-Mediated Stress Response

ET can promote fruit ripening and leaf senescence, induce adventitious roots and root hairs, break the dormancy of plant seeds and buds, and inhibit flowering in many plants (but induces and promotes flowering in pineapple and its congeners). Ethylene, one of the six plant hormones, also plays a role in a variety of stress responses, such as salt, low temperature, and flooding [75]. AP2/ERF transcription factor *submergence 1A* (*Sub1A*) is a rice flooding tolerance gene, the expression of which was up-regulated under flooding conditions, while ET treatment induced its expression, but GA treatment did not. Flood-tolerant rice varieties containing the *Sub1A* gene synthesised ET in the plants under flooded conditions, and ethylene promoted the degradation of ABA on the one hand and the expression of *Sub1A* on the other [76,77]. The ethylene-responsive transcription factor RAP2-2 (RAP2.2) in *Arabidopsis* belongs to the same subfamily as *Oryza sativa* submergence tolerance gene *Sub1A*. RAP2.2 is induced by ET in shoots and functions in ET-controlled signal transduction pathways, and overexpression of *RAP2.2* lines showed increased survival under hypoxia stress [78].

### 3.5. AP2/ERF Transcription Factors Involved in BR-Mediated Stress Response

BR has an important role in plant growth and development and, together with other plant hormones, is involved in the regulation of many aspects of plant development, including stem and leaf growth, root growth, vascular tissue differentiation, fertility, seed germination, maintenance of apical dominance, and plant photomorphogenesis [79]. Liu et al. [80] demonstrated that *AP2/ERF family transcription factor 72* (*ERF72*) may be a candidate gene for cross-interaction between the BR signalling pathway and stress response. In *Arabidopsis*, ERF72/RAP2.3 antagonised BZR1 (brassinazole-resistant 1) and ARF6 (Auxin responsive factor 6) and inhibited hypocotyl elongation. Schmitz et al. [81] found SUB1A differentially regulates the expression of BR synthesis-related genes during plant flooding, activates BR biosynthesis and signal transduction, induces the expression of *Gibberellin 2-oxidase 7* (*GA2ox7*), a key gene for GA degradation, and, thus, controls GA levels in rice plants.

### 3.6. AP2/ERF Transcription Factors Involved in CTK-Mediated Stress Response

The main physiological role of CTK is to promote cell division and prevent leaf senescence. The senescence and yellowing of green plant leaves occur due to the decomposition of proteins and chlorophylls within them. At the same time, CTK can maintain protein synthesis, thus keeping the leaves green and promoting the differentiation of buds, cell enlargement, the development of lateral buds and the removal of apical dominance [82]. Moreover, CTK also plays an important role in plant response to abiotic stresses [83]. AP2/ERF-N22(2) has an AP2 structural domain consisting of 55 amino acid residues and a set of acidic amino acid residues in the C-terminal region that can act as a trans-activating structural domain. Moreover, AP2/ERF-N22(2) belongs to group VI L. Members of *Arabidopsis* group VI L have been shown to be involved in the response to cytokinins under drought stress [84]. Cytokinin response factor (CRF) is an AP2/ERF family transcription factor that regulates plant cotyledon and embryo developmental processes and is also involved in cytokinin signalling [85,86]. In *Arabidopsis*, cytokinin response factor 6 (CRF6) is an AP2/ERF transcription factor that is induced by CTK. *CRF6* is highly expressed in the veins of mature leaves, promotes CTK synthesis, and is induced by abiotic stress [87,88]. Zwack et al. [89] showed that cytokinin response factor 4 (CRF4) can be induced by cold exposure for a short period of time, especially in root and shoot tissues. Altered transcriptional expression of genes of the cold signalling pathway in c-repeat binding factors (CBF) and cold-regulated 15a (COR15a) in *crf4* mutants and overexpression *CRF4* lines suggests that CRF4 may be involved in this pathway. The specific regulatory mechanism of CRF is still unclear, so future identification of CRF target genes and upstream signalling molecules could help explain this.

## 4. Role of AP2/ERF Transcription Factors in Response to Abiotic Stresses (Not Dependent on Hormone Signalling Pathway)

Stresses such as drought, high salt, high temperature, low temperature, nutrients, and heavy metals are the major abiotic stress factors affecting plant growth and development, crop yield, and quality. In the course of plant evolution, complex defence mechanisms have been developed to adapt to abiotic stresses so that plants can improve their ability to tolerate abiotic stresses. Studies have shown that the AP2/ERF family of transcription factors is widely involved in regulating the plant response to various abiotic stresses [90].

### 4.1. AP2/ERF Transcription Factors in Response to Drought Stress

Water is a vital resource for the survival of all life and has played an important role in the evolution of life. Water is an essential constituent for photosynthesis in green plants, and if there is a lack of water, the plant’s photosynthesis will be weakened. Leaves will wilt and, in severe cases, can lead to the death of the plant [91]. Yu et al. [92] found that overexpression of the AP2/ERF family gene TaERF-6-3A increased sensitivity to drought and salt stress in *Arabidopsis*. Expression of stress-related and antioxidant-related genes was down-regulated in overexpressing *TaERF-6-3A* plants. Genome-wide analysis of the AP2/ERF genes in *Pisum sativum* (L.) identified 153 AP2/ERF genes. Jarambasa et al. [93] found that *DREB2A*, *DREB2C*, *DREB2E*, and *DREB2F* were induced in leaves under drought stress. Kumar et al. [94] showed that overexpressing the *OsAP2/ERF-N22* line showed higher relative water content, membrane stability index, wax content, osmotic potential, stomatal conductance, and transpiration rate activities. Kabir et al. [95] demonstrate that a total of 119 CoAP2/ERF genes were identified from the dark jute genome. *CoDREB-11*, *CoDREB-14,* and *CoRAV-01* genes were significantly up-regulated under salinity and drought stress conditions. Kong et al. [96] demonstrate that the osmotic stress-induced PtoERF15 and its target gene *PtoMYC2b*, which is involved in mediating blood vessel size, density, and cell wall thickness in response to drought in poplar, were identified and characterised. Overexpression of *PtoERF15* contributes to the maintenance of stem water potential, thereby increasing drought tolerance.

### 4.2. Molecular Mechanisms of AP2/ERF Associated with Salt Stress

Soil salinity affects around 6% of the world’s land and 23% of arable land, causing considerable economic losses through crop stress and reduced yields. Because salinity plays a vital role in plant growth, excess soluble salts will have a toxic effect on plants above a certain limit; this can greatly affect the growth and development of plants, ultimately resulting in reduced yields [97]. AP2/ERF transcription factor 19 (OsERF19) was identified in rice by Huang et al. [98]. The *OsERF19* expression was inhibited by salt stress. Overexpressing the *OsERF19* line increased the tolerance of plants to salt stress. In addition, *Late embryogenesis abundant protein gene* (*OsLEA3*), *Vacuolar Na^+^/H^+^ antiporter gene* (*OsNHX1*), *Low-affinity Na^+^ transporter* (*OsHKT6*), and *Overly tolerant to salt 1* (*OsOTS1*) genes were up-regulated in overexpressing lines when plants were subjected to salt stress. Within the AP2/ERF family, transcription factor 71 (AtERF71)/HRE2 is known to be involved in hypoxia and osmotic stress responses [99,100]. Seok et al. dissected the *HRE2* promoter and showed that the -116 to -2 region is responsible for hypoxia and salt stress responses. This region contains both positive and negative cis-regulatory elements that may regulate *HRE2* expression under salt stress [101].

### 4.3. AP2/ERF Transcription Factors Involved in Plant Response to Temperature Stress

#### 4.3.1. AP2/ERF Transcription Factors and High Temperature Stress

**Figure 2:**
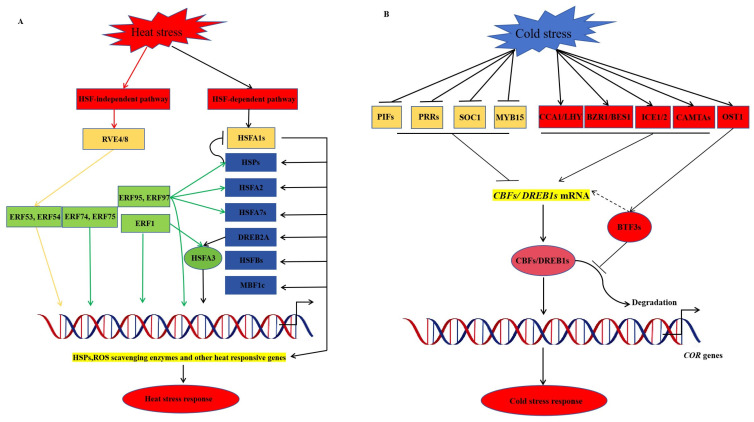
AP2/ERF transcription factor-mediated temperature stress response model in plants. (**A**) The AP2/ERF transcription factor-mediated heat stress response model. In the HSF-independent pathway, REVEILLE4 and 8 (RVE4/8) are the major transcription factors that regulate the downstream expression of ERF53 and ERF54 and mediate plant heat tolerance. In the HSF-dependent pathway, heat stress induces the expression of *HSFA1*, which is a master regulator of transcriptional regulation. Under non-stress conditions, heat-shock proteins (HSPs) repress *HSFA1* expression, such as *HSFA2*, *HSFA7*, *DREB2A*, *HSFBs*, and *multiprotein bridging factor 1c* (*MBF1c*), which are involved in a key transcriptional regulatory cascade. DREB2A further activates *HSFA3*, which activates or fine-tunes the expression of *HSPs*, ROS scavenger enzymes, and other *HSR* gene expressions. (**B**) The AP2/ERF transcription factor-mediated cold stress response model. Cold stress induces the expression of *C repeat binding factor*/*dehydration response element binding protein 1s* (*CBF/DREB1s*). *CBFs/DREB1* genes are regulated by multiple transcription factors and integrate multiple signalling pathways. The light signalling components Phytochrome-interacting factors (PIF3, PIF4, and PIF7), the circadian oscillator component Pseudo response regulator (PRR9, PRR7, and PRR5), as well as other transcription factors such as Suppressor of overexpression of constans 1 (SOC1) and MYB transcription factor MYB15, negatively regulate *CBFs/DREB1* gene expression. The genes CCA1, LHY, BR, BZR1/BES1, ICE1, ICE2, CAMTA, and other transcription factor inducers directly enhance the expression of CBF/DREB1 by binding to its promoter region. The protein stability of CBF/DREB1 is positively regulated by basic transcription factor 3 (BTF3) phosphorylated by Open stomata 1 (OST1). AP2/ERF transcription factor ERF regulates *CBF/DREB1* expression by binding directly and indirectly to the CBF/DREB1 promoter. ERF also directly regulates the expression of other *COR* genes by binding to their promoters. Arrow ends indicate the activation effect; bar ends indicate the repression effect.

Temperature is the main environmental factor affecting plant growth and development and the quality of life of the fruit after harvest; appropriate temperatures promote plant growth. High temperatures primarily harm plant growth and development, shorten the plant, cause localised burns on the leaves and stems, and reduce the number of flowers. High temperatures also encourage increased transpiration, disrupting the water balance and causing the plant to wilt and die [102]. Zhang et al. [103] found that the AP2/ERF transcription factor PlTOE3 can specifically activate the *Tryptophan decarboxylase gene* (*PlTDC*) promoter. High-temperature stress can affect the transcriptional level of PlTOE3. Overexpression of *PlTOE3* in tobacco enhanced plant melatonin production and heat stress tolerance, whereas silencing of *PlTDC* expression gave the opposite results. The SHN/WIN evolutionary branch of the AP2/ERF transcription factor family is involved in many important processes. In fact, plants respond to heat stress through heat shock transcription factor (HSF) and heat shock protein (HSP)-mediated heat stress response (HSR), and some *NSR* genes can be activated in heat-activated stress. Knockout of *heat shock transcription factor A1* (*HSFA1*) gene plants results in reduced activation of a large number of *HSR* genes and a heat stress-sensitive phenotype [42,104]. The *Arabidopsis* genome encodes 21 *HSFs*, which can be classified into three categories: *HSFA*, *HSFB*, and *HSFC* [105]. *HSFA2* is the most heat-inducible HSF; its heat induction is dependent on HSFA1 [106]. The *HSFA2* knockout plants exhibit a significant heat-sensitive phenotype [107]. AP2/ERF transcription factor DREB2A acts downstream of HSFA1 and is another important thermotropic transcriptional activator. Overexpression of a constitutively active variant of *DREB2A* up-regulates *HSR* genes in *Arabidopsis* [108,109]. Under heat stress, AP2/ERF transcription factor ERF1 positively regulates heat tolerance in plants by binding to DRE cis-elements in heat-responsive genes, such as *HSFA3* and *HSPs*, and activating their expression [110]. Ectopic constitutive expression of *ERF95* or *ERF97* enhances basal heat tolerance in plants. AP2/ERF transcription factor ERF95 interacts with ERF97, and this interaction is enhanced at high temperatures, where they directly regulate a common transcriptional regulator, *HSFA2,* in response to heat stress [111]. REVEILLE4 (RVE4) and RVE8 play important roles in regulating heat tolerance in plants. The *rve4* and *rve8* double mutants are more susceptible to high temperatures. RVE4/8 regulates heat tolerance by a mechanism that is independent of hsfa1-mediated HSR and is partially influenced by the downstream transcription factors ERF53 and ERF54 [112]. RESPIRATORY BURST OXIDASE HOMOLOG D (RbohD) is an NADPH oxidase that contributes to the production of ROS. These ROS molecules act as signalling molecules to initiate heat stress responses and transduction. AP2/ERF transcription factors ERF74 and ERF75 regulate RbohD transcription in response to heat stress [113] (Figure 2A).

#### 4.3.2. AP2/ERF Transcription Factors and Low Temperature Stress

Symptoms of low temperature are discolouration of plant leaves, necrosis, and the appearance of spots on the surface of the plant, making the plant grow slowly and inducing other changes in morphological characteristics; the photosynthetic rate is significantly reduced, resulting in lower yields and lower quality. However, low-temperature stress not only leads to a reduction in plant yield, but in severe cases, it can also cause plant death [114]. Ren et al. [115] identified the apetala/ethylene responsive factor (AP2/ERF) family apetala 2.4 (RAP2.4) in *Chrysanthemum lavandulifolium* and plays an important role in plant development and response to stress. The activities of superoxide dismutase, peroxidase, and proline content in leaves in the four overexpression lines were higher than those in the wild type (WT). In contrast, the electrical conductivity and malondialdehyde content were decreased, indicating that the tolerance of plants with *ClRAP2.4* overexpression to cold stress was increased. The SOD and POD activities, as well as proline content, were higher in the overexpressing *ClRAP2.4* strain than in the wild type, whereas conductivity and malondialdehyde content were reduced, suggesting that overexpression of *ClRAP2.4* is increased in plants tolerance to cold stress. Heidari et al. [116] treated two tomato species with low temperatures and found increased ABA content in two tomato species but increased ZT content in cold-tolerant tomato species. The contents of IAA and GA in cold-sensitive tomato species are reduced by low temperatures. The CTK was also found to be an important plant hormone associated with low-temperature stress in tomatoes. They also found that the *C-repeat/DRE binding factor 1* (*CBF1*) gene is less induced in response to low temperature in tomato, but transcription of the inducer of the *CBF expression 1* (*ICE1*) gene was up-regulated under low temperature in both tomato species. ICE1 appears to regulate *cold-regulated* (*COR*) genes in a manner that is not dependent on CBF. Numerous studies have shown that ethylene signalling regulates freezing tolerance in plants by repressing *C repeat binding factor* (*CBF*) motifs [117]. The *CBF* genes are regulated by several transcription factors, such as Phytochrome-interacting factors (PIF3, PIF4, and PIF7), which are light signalling components, whereas Pseudo response regulators (PRR9, PRR7, and PRR5) are circadian oscillatory components that negatively regulate the expression of *CBFs* [118,119,120,121]. Circadian clock associated 1 (CCA1), late slender hypocotyl (LHY), and brassinosteroids (BRs) signalling pathway components BRASSINAZOLE-RESISTANT (BZR1)/BRI1-EMS-SUPPRESSOR1 (BES1) positively regulate the expression of *CBFs* by directly binding to the promoter region and increasing the freezing tolerance of the plant [122]. There are other transcription factors involved in cold-induced expression of *CBFs*, such as suppressors of overexpression of constant 1 (SOC1) and MYB transcription factor MYB15, which are negative regulators. In contrast, inducers of CBF expression (ICE1, ICE2) and calcium-binding activators of transcription (CAMTAs) act as positive regulators [123,124] (Figure 2B).

### 4.4. Role of Plant AP2/ERF in Response to Nutritional Element Stress

Nitrogen (N), phosphorus (P), and potassium (K), which are essential nutrients for plants, play very important physiological roles in plant growth and development. N is found in both proteins and nucleic acids, and proteins, in turn, are the basic substances that make up protoplasm [125].

N is also a constituent of chlorophyll, which is indispensable for photosynthesis in higher green plants and is, therefore, essential for plant photosynthesis [126]. RNA-seq analysis by Joshi et al. [127] showed that N stress induced most of the transcriptome changes in spinach roots, identifying 1,346 differential expressed genes (DEGs). In the presence of high N in leaf tissues, a subset of AP2/ERF family member transcription factors were all overexpressed in tissues in response to N perturbation. Bacterial accommodation within living plant cells was restricted to nitrogen-fixing rhizobium symbiosis; bacterial uptake is mediated by tubular structures called infection threads. Cerri et al. [128] identified a gene encoding an AP2/ERF transcription factor known as ethylene-responsive transcription factor (ERN1). *Rhizobium primordia* were formed following mutation of the *ern1* gene in *Lotus japonicus*, but the majority remained uninfected, and bacterial entry into the root epidermis via infection threads was eliminated.

P is a constituent of the nucleus and nucleic acids, which have a special role in plant life and hereditary processes [129]. Most terrestrial plants establish symbiotic relationships with arbuscular mycorrhizal fungi (AMF), providing them with lipids and sugars in exchange for phosphorus and nitrogen. Zhang et al. [130] identified the AP2/ERF transcription factor mycorrhisation 1 (MtERM1) as being able to bind directly to the AW-box and AW-box-like cis-elements in the two half-size ABCG transporters (MtSTR2 and MtSTR) promoters, which are required for host lipid efflux and tuft development. Chen et al. [131] identified that the AP2/ERF transcription factor gene *PalERF2* overexpression lines enhanced tolerance to Pi deficiency. In addition, overexpression of *PalERF2* up-regulated phosphate starvation-induced (PSI) gene expression level and increased phosphate uptake under drought conditions.

K is an activator of many of the enzymes of photosynthesis and enhances the activity of the enzymes and, hence, promotes photosynthesis. K also functions to control the opening and closing of the plant’s stomata and hence facilitates the conservation of water by the plant [132]. Kim et al. [133] showed that the AP2/ERF family transcription factor RAP2.11 was identified as a component of the low potassium response. The AP2/ERF family transcription factor RAP2.11 regulates the expression of the *high-affinity K^+^ uptake transporter protein* (*AtHAK5*) under low K^+^ conditions and also contributes to a coordinated response to low potassium conditions by regulating other genes in the low K^+^ signalling cascade. Chen et al. [134] demonstrated that AP2/ERF family transcription factor *OsERF106* was expressed in germinating seeds, primary roots, and developing flowers. Overexpression of *OsERF106* resulted in stunted growth, relatively high levels of malonic dialdehyde (MDA) and reactive oxygen species (ROS), reduced CAT activity, and excessive accumulation of sodium (Na^+^) and potassium (K^+^) ions in transgenic rice.

### 4.5. AP2/ERF Involved in Plant Response to Heavy Metals Stress

Heavy metals in soil are a stress factor for plants, affecting their survival in various ways, including growth, development, and reproduction. Excessive amounts of heavy metals affect photosynthesis in plants, thus causing symptoms such as yellowing and wilting of plant leaves and reducing the efficiency of light energy utilisation in plants. High concentrations of heavy metals in the soil affect the ability of plants to absorb and utilise nutrients, leading to a reduction in the number of leaves, changes in the root system, and a reduction in stem expansion [135,136,137,138]. Karanja et al. [139] identified a total of 247 ERF family genes in the radish genome, and a portion of the AP2/ERF genes were preferentially expressed under drought and heat stress, whereas they were repressed under heavy metal stress. Chen et al. [140] identified a large number of key heavy metal Cd-induced DEGs containing transcription factors such as AP2/ERF, MYB, NAC, and WRKY. DEGs are involved in antioxidants, heavy metal transport, and detoxification pathways, and AP2/ERF family transcription factors were suggested to play crucial roles in kenaf Cd tolerance. Tian et al. [141] conducted a study exposing *Solanum tuberosum* L. to heavy metals Cd/Pb/Zn/Ni/Cu. They identified 181 potential StAP2/ERF genes, and the *StAP2075*, *StAP2077,* and *StAP2126* genes were found to promote Cd accumulation and yeast growth (Cd detoxification phenotype).

## 5. Conclusions and Prospects

**Figure 3:**
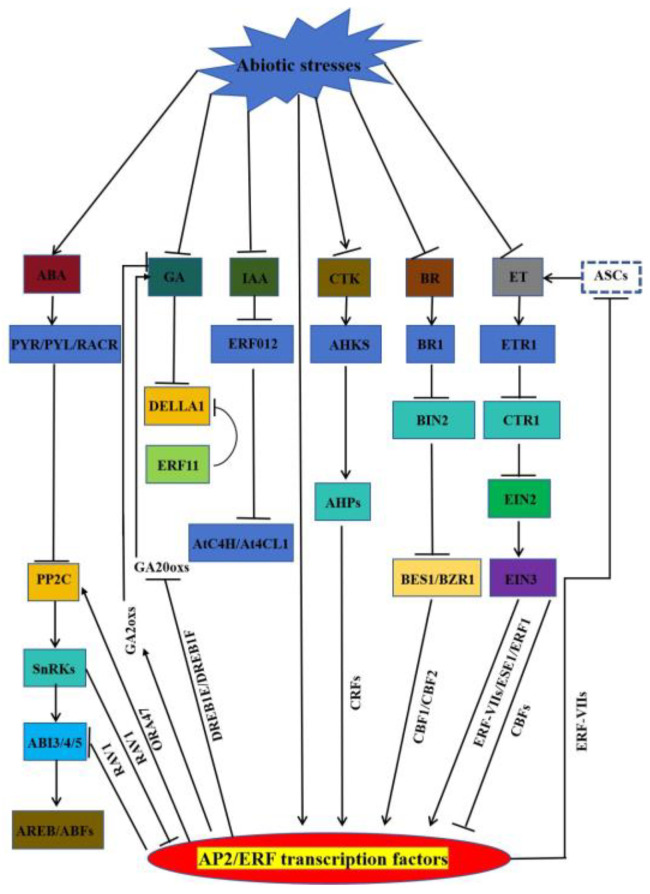
AP2/ERF transcription factor participates in the regulation of hormone-mediated response networks during abiotic stresses. Abiotic stress can alter the production and distribution of plant hormones and subsequently mediate stress responses through AP2/ERF family transcription factors and hormone signalling components. ABA: abscisic acid; GA: gibberellin; IAA: auxin; BR: oleuropein lactone; CTK: cytokinin; ET: ethylene. Arrow ends indicate the activation effect; bar ends indicate the repression effect.

Nowadays, there are many reports on the involvement of AP2/ERFs in the regulation of hormone signalling-mediated stress response. When a plant suffers from abiotic stress, the inducing hormones (ABA and ET) and growth-promoting hormones (GAs, IAA, CTK, and BRs) carry out their defence against the adverse external environment through a mechanism regulated by AP2/ERFs (Figure 3) [142].

ABA Pathway: Under abiotic stress conditions, when plants are subjected to water deficit, the rate-limiting ABA biosynthetic enzyme Nine-cis-Epoxycarotenoid Dioxygenase (NCED) is rapidly up-regulated to promote ABA biosynthesis [143]. Subsequently, ABA is sensed by ABA receptors (PYR/PLY/RCAR), which form a ternary complex with protein phosphatase 2Cs (PP2Cs) as a ternary complex, resulting in the removal of the inhibition of SnRK2 kinases (SnRK2s). Active SnRK2 phosphorylates downstream substrate proteins, including AREBs/ABFs, ion channels, and nicotinamide adenine dinucleotide phosphate (NADPH) oxidases, thereby inducing ABA response [144,145]. ERF18/ORA47 activates the PP2C family phosphatase gene *ABI2*. Meanwhile, ABI1 acts upstream of ORA47 to activate ORA47, forming an ABI1-ORA47-ABI2 regulatory loop that inhibits ABA signalling and drought tolerance [39]. RAV1 inhibits root growth sensitivity to ABA by repressing *ABI3*, *ABI4*, and *ABI5* expression. SnRK2.2, SnRK2.3, and SnRK2.6 also interact and phosphorylate RAV1 to inhibit transcriptional repression of target genes [146]. GA Pathway: In the absence of GA, DELLA inhibits GA responses. Abiotic stress leads to reduced GA content and signalling by inhibiting AP2/ERF-mediated GA biosynthesis enzymes. DREB1E and DREB1F lead to salt stress-induced growth retardation mainly by inhibiting *GA20oxes* [147]. Overexpressing *CBF1* and *ERF6* plants are sensitive to stress-induced growth retardation due to increased expression of *GA2oxs* and accumulation of DELLA. ERF11 promotes plant internode elongation by activating GA biosynthesis, and the expression of *GA3ox1* and *GA20oxs* is increased in overexpressing *ERF11* plants [148]. IAA Pathway: Huang et al. found that overexpression of *ERF012* showed resistance to temperature, drought, salt, and heavy metal stresses. Overexpression of *ERF012* inhibited root growth and promoted root hair development and leaf senescence. The application of exogenous IAA effectively mitigated this effect. ERF012 may down-regulate its target genes, *AtC4H* and *At4CL1* (key genes for phenylpropanoid metabolism and cell wall formation), resulting in reduced IAA accumulation [73]. CTK Pathway: More than half of the CTK-responsive genes are regulated by both CRF and B-type ARRs (typical cytokinin-responsive transcription factors), and CRF6 also cooperates with CTK signalling to inhibit stress-induced leaf senescence through a common subset of CTK-regulated genes. CRF6 also represses CTK-related target genes involved in CTK biosynthesis, signalling, and transport to mitigate the adverse effects of CTK on abiotic stresses [87,88]. BR Pathway: BR is detected by the plasma membrane receptor kinase BRASSINOSTEROID INSENSITIVE 1 (BR1), which represses the activity of the negative regulator BRASSINOSTEROID INSENSITIVE 2 (BIN2), leading to the accumulation of the transcription factor BRASSINAZOLE-RESISTANT 2/BRI1-EMS-SUPPRESSOR 1 (BES1/BZR1), which is responsible for regulating the genes involved response to plant growth and stress responses in relation to BR. BR positively regulates cold tolerance partly through the CBF-mediated cold response pathway, in which BZR1 binds and promotes *CBF1*/*CBF2* expression in response to cold. Cold stress also promotes the accumulation of the unphosphorylated active form of BZR1 through unknown mechanisms [149,150]. ET Pathway: ET is synthesised by the rate-limiting enzyme ACC synthase (ACS), a major target for regulating ET production under stress conditions [151]. The binding of ET to its receptor ethylene-insensitive 1 (ETR1) inactivates constitutive triple response 1 (CTR1) kinase activity, releasing CTR1 from its inhibition of ethylene-insensitive 2 (EIN2). The C-terminus of EIN2 then translocates to the nucleus, activating ethylene insensitive 3 (EIN3) and the transcriptional cascade of ethylene-regulated genes [152,153]. ET represses *CBF* to regulate cold stress negatively, positively regulates ERF-VII-mediated flooding and inundation, and enhances salt tolerance by activating *ERF1* and *ESE*. The ET-insensitive mutants *etr1*, *ein2,* and *ein3* show enhanced freezing tolerance. EIN3 represses *CBF* expression by directly binding to its promoter. Flooding causes hypoxia, which promotes ET production and activates the expression of a group of ERF-VIIs; however, ERF-VIIs regulate the hypoxic response partly through an ET-independent pathway. ERF-VII may also negatively regulate ET signalling and homeostasis through feedback regulation [154]. The involvement of AP2/ERFs in the regulation of hormone signalling-mediated stress response has been widely reported. However, we do not know the exact regulatory mechanism; more scientific exploration is needed. Question 1: How does AP2/ERF feedback regulate hormone biosynthesis and metabolism when they receive hormone signals? Question 2: How do AP2/ERFs regulate the expression of downstream related genes by synergising or antagonising multiple hormone signal transduction groups? Question 3: Can AP2/ERFs interact with other family transcription factors to jointly regulate the expression of downstream related genes through hormone signal transduction? Explaining these three issues will help to systematically explain the regulatory network of AP2/ERFs in response to abiotic stress conditions in plants and provide a theoretical basis for subsequent plant stress tolerance breeding projects.

Plants respond to abiotic stress not only through the above hormone signalling networks but also through a series of other complex signalling pathways and gene expression regulatory networks that have been established during the long-term evolution of plants. When plants are affected by external stress, they are able to regulate their growth and development and their ability to cope with tolerance under abiotic stress. AP2/ERF family transcription factors can play a role in abiotic stress response through hormone-dependent or hormone-independent signalling pathways. In recent years, there have been many reports on the involvement of AP2/ERF family transcription factors in plants’ response to abiotic stresses (Table 1). But they are still focused on abiotic stresses, such as drought, high salt, high temperature, and low temperature; the molecular mechanisms of AP2/ERF transcription factors in response to chemical reagent stress are rarely reported, for example, pesticides, car exhaust, and haze. Car exhaust and haze, the main component of which is sulphide, can cause plants to develop leaf scorch and impede growth and development. Excessive use of pesticides can damage the ecosystem and lead to a decline in crop yields. Therefore, it is important to study AP2/ERF in response to chemical stress in plants. Our in-depth study of the molecular mechanisms of plant responses to abiotic stresses, specifically the AP2/ERF transcription factors, encompasses various aspects such as stress signal perception and transmission, transcriptional regulation, and expression of response genes. This research aims to guarantee plants’ normal growth and development under abiotic stresses, thereby ensuring food production. Ultimately, this contributes to global food security and enhances the quality of human life.

## Figures and Tables

**Table 1:** Abiotic stress responsive AP2/ERF transcription factors in plants.

Abiotic Stress Type	AP2/ERF Transcription Factors	Species	Reference
Drought	*AtDREB1A*	*Arabidopsis thaliana* L.	[155]
Cold	*DREB1/CBF*	*Arabidopsis thaliana* L.	[156]
Cold	*DREB2*	*Arabidopsis thaliana* L.	[156]
Cold, salt, drought	*GmDREBa*	Soybean (*Glycine max* L.)	[157]
Cold, salt, drought	*GmDREBc*	Soybean (*Glycine max* L.)	[157]
Drought	*ERF1-V*	Wheat (*Triticum aestivum*)	[158]
Temperature	*ZjDREB1.4*	Zoysiagrass (*Zoysia japonica* S.)	[159]
Cold, salt, drought	*ZmEREB3*	Maize (*Zea mays* L.)	[160]
Salt	*ZmEREB20*	Maize (*Zea mays* L.)	[70]
Drought	*ZmEREB46*	Maize (*Zea mays* L.)	[161]
Drought	*ZmEREB60*	Maize (*Zea mays* L.)	[162]
Drought	*ZmEREB137*	Maize (*Zea mays* L.)	[163]
Waterlogging	*ZmEREB180*	Maize (*Zea mays* L.)	[164]
Osmotic	*ZmEREB204*	Maize (*Zea mays* L.)	[165]
Drought	*ZmEREB240*	Maize (*Zea mays* L.)	[166]
Cold	*OsDREB1A*	Rice (*Oryza sativa*)	[167]
Temperature	*OsDREB1B*	Rice (*Oryza sativa*)	[168]
Cold, salinity	*OsDREB1D*	Rice (*Oryza sativa*)	[169]
Drought	*OsDREB1E*	Rice (*Oryza sativa*)	[169]
Drought	*OsDREB1G; OsDREB1I*	Rice (*Oryza sativa*)	[69]
Drought	*OsDREB2B*	Rice (*Oryza sativa*)	[69]
Salt, drought, temperature	*OsDREB4-1*	Rice (*Oryza sativa*)	[170]
Salt, drought, temperature	*OsDREB1F*	Rice (*Oryza sativa*)	[171]
Temperature	*OsWR2*	Rice (*Oryza sativa*)	[172]
Drought	*OsERF71*	Rice (*Oryza sativa*)	[173]
Drought	*OsLG3; OsERF62; OsRAF*	Rice (*Oryza sativa*)	[174]
Drought	*OsAP37*	Rice (*Oryza sativa*)	[175]
Salt	*OsAP23*	Rice (*Oryza sativa*)	[176]
Salt, drought, temperature	*OsEREBP2*	Rice (*Oryza sativa*)	[177]
Salt	*SERF1*	Rice (*Oryza sativa*)	[178]
Salt	*OsIDS1*	Rice (*Oryza sativa*)	[179]
Salt	*OsERF922*	Rice (*Oryza sativa*)	[180]

## Data Availability

Not applicable.

## Supplementary Materials

The following supporting information can be downloaded at https://www.mdpi.com/article/10.3390/ijms25020893/s1.
